# Transgenic expression of Notch-1 intracellular active domain (N1IC) in T cells: a potential therapy to overcome suppression induced by tumors

**DOI:** 10.1186/2051-1426-1-S1-O13

**Published:** 2013-11-07

**Authors:** Rosa A Sierra, Dorota Wyczechowska, Paulo C Rodriguez

**Affiliations:** 1 Stanley S. Scott Cancer Center, Louisiana State University, New Orleans, LA, USA

## 

An impaired anti-tumor immunity is found in patients with cancer and represents a major obstacle in the successful development of different forms of immunotherapy. Several pathways have been described to explain the induction of T cell anergy in tumors; however, the translation of this understanding into new adjuvants for immunotherapy has been limited. Pharmacological inhibition of Notch-homolog-related proteins (Notch) in T cells leads to a similar inhibitory effect to that found in tumors. Thus, we aimed to determine the role of Notch in the function of T cells and to test the effect of the transgenic expression of Notch 1 intracellular active domain (N1IC) in a tumor model of T cell-based immunotherapy. Our results show an increased expression of Notch 1 and 2, but not Notch 3 and 4, in activated CD4+ and CD8+ T cells. In addition, conditional deletion of both Notch 1 and 2 in antigen-specific CD4+ and CD8+ T cells, but not individual deletion of Notch forms, prevented cell proliferation and IFNγ production. Interestingly, tumor-associated myeloid-derived suppressor cells (MDSC) inhibited the expression of Notch 1/2 in activated T cells at the same ratios at which they blocked T cell proliferation and IFNγ production. To address the relevance of the decreased expression of Notch in T cells in tumor-induced tolerance, we adoptively transferred antigen-specific CD8+ T cells over-expressing N1IC (OT-1 N1ICf/f Gnz-Cre+/-) or floxed controls, into mice bearing established 3LL tumors carrying the model antigen ovalbumin (OVA). A higher anti-tumor effect and IFNγ production was observed in tumor-bearing mice transferred with N1IC CD8+ cells, as compared to those receiving floxed cells. Then, we tested whether N1IC directly promoted T cell cytotoxicity. An increased antigen-specific killing against OVA-loaded cells was induced by N1IC CD8+ cells in vitro and in vivo, as compared to control cells. This effect correlated with an increased expression in N1IC CD8+ T cells of the degranulation marker CD107a and the effector molecules granzyme B and IFNγ. Interestingly, N1IC endogenously bound to granzyme B promoter, suggesting a direct role of N1IC in the induction of effector genes in T cells. Moreover, an increased expression of T cell survival receptors CD122, IL-7R, and CD44 was found in N1IC mice, suggesting a potential role of Notch signaling in T cell survival/stemness. Altogether, these results suggest the role of Notch-1/2 in T cell function and the potential use of N1IC as an adjuvant for T cell-based immunotherapy. Continuation of this work could enable the design of new therapeutic approaches to reverse T cell anergy in individuals with cancer.

